# Targeting mitochondrial apoptosis in septic lung injury: protective potential of omega-3 fatty acids

**DOI:** 10.1007/s10495-026-02325-y

**Published:** 2026-03-27

**Authors:** Ersin Çelik, Pınar Karabacak, Mustafa Soner Özcan, İlter İlhan, Muhammet Yusuf Tepebaşı, Emine Sarman, Melih Arlıoğlu, Halil Aşcı

**Affiliations:** 1 Department of Anesthesiology and Reanimation, Faculty of Medicine, Suleyman Demirel University, Cunur, Isparta, Turkey; 2 Department of Biochemistry, Faculty of Medicine, Suleyman Demirel University, Isparta, Turkey; 3 Department of Genetic, Faculty of Medicine, Suleyman Demirel University, Isparta, Turkey; 4 Department of Histology and Embryology, Faculty of Medicine, Afyonkarahisar Health Sciences University, Afyonkarahisar, Turkey; 5 Department of Pharmacology, Faculty of Medicine, Suleyman Demirel University, Isparta, Turkey

**Keywords:** Omega-3 fatty acids, Acute lung injury, Mitochondrial apoptosis, Lipopolysaccharide, Sepsis

## Abstract

Acute lung injury secondary to sepsis remains a major problem in critical patients. Lipopolysaccharide (LPS)-induced lung injury is characterized by inflammatory infiltration, oxidative stress, and extensive apoptosis, particularly via the mitochondrial pathway. Omega-3 (OM3) polyunsaturated fatty acids have been proposed as promising modulators of inflammation and oxidative damage. However, their role in regulating mitochondria-mediated apoptosis in sepsis remains underexplored. Thirty-two female Wistar albino rats were randomized into four groups: Control, LPS (5 mg/kg, i.p. 3th), LPS-OM3 (400 mg/kg/day, i.p., 3 day), and OM3 only. After 72 h, lung tissues were collected for histopathological scoring, immunohistochemistry as tumor necrosis factor-alpha (TNF-α), heat shock protein (HSP70), caspase-3 (Cas-3); biochemical analyses as total oxidant status (TOS), total antioxidant status (TAS), oxidative stress index (OSI) and qRT-PCR for mitochondrial apoptosis markers B cell lymphoma-2 (Bcl-2), Bcl-2-associated X protein (Bax), cytochrome-c (Cyt-c) and Cas-9. LPS induced ALI characterized by bronchiolar epithelial degeneration, alveolar hemorrhage, inflammation, and hyaline membrane formation. Biochemically, TOS and OSI levels were significantly elevated, while TAS remained unchanged. Immunohistochemical and genetic analysis showed marked upregulation of TNF-α, HSP70, and Cas-3 Bax, Cyt-c, and Casp-9 expression and decreased Bcl-2 expression in the LPS group. OM3 supplementation significantly attenuated histopathological damage, reduced oxidative stress markers, suppressed immunopositivity of proinflammatory and apoptotic proteins, and favorably modulated the apoptosis-related gene expressions. OM3 fatty acids exert a potent protective effect against LPS-induced ALI by suppressing mitochondrial apoptosis, reducing oxidative stress, and modulating the inflammatory response. These findings highlight the therapeutic potential of OM3 in mitigating sepsis-associated pulmonary damage through mitochondrial preservation.

## Introduction

Acute lung injury (ALI) and its more severe form, acute respiratory distress syndrome (ARDS), remain critical complications of systemic inflammatory states such as sepsis, with high morbidity and mortality rates worldwide. Among all organs affected by sepsis, the lung is particularly vulnerable due to its vast microvascular network and extensive exposure to circulating inflammatory mediators. Lipopolysaccharide (LPS), a potent endotoxin derived from Gram-negative bacteria, is commonly used in experimental models to mimic sepsis-induced lung injury. It provokes a cascade of events including the release of proinflammatory cytokines, oxidative stress, and ultimately cellular apoptosis, all of which contribute to alveolar-capillary barrier dysfunction and impaired gas exchange [15].

While inflammation and oxidative stress have long been recognized as central components of ALI pathophysiology, increasing attention has turned toward the role of mitochondrial dysfunction and apoptosis in the progression of lung injury. Mitochondria, beyond their role in ATP production, are integral to cellular fate decisions and immune responses. In the context of LPS-induced stress, mitochondrial membranes become permeable due to the activation of pro-apoptotic B-cell lymphoma 2 (Bcl-2) family members such as Bcl-2-associated X protein (Bax), leading to the release of cytochrome c (Cyt-c) and subsequent activation of caspase (Cas)-9 and Cas-3, hallmark events of intrinsic apoptosis [9, 23]. Excessive apoptosis, particularly of alveolar epithelial and endothelial cells, exacerbates pulmonary edema, increases alveolar permeability, and disrupts lung architecture, hallmarks of ALI and ARDS [24].

The contribution of mitochondrial apoptosis to lung tissue destruction is further intensified by oxidative stress, as reactive oxygen species (ROS) not only damage lipids and DNA but also act as secondary messengers that amplify apoptotic signaling. In this context, oxidative burden reflected by elevated total oxidant status (TOS) and oxidative stress index (OSI) further destabilizes mitochondrial membranes, creating a self-perpetuating cycle of injury [12]. Thus, interventions that target both mitochondrial integrity and oxidative balance hold promise as dual-action therapies in ALI.

Among such candidates, omega-3 (OM3) polyunsaturated fatty acids (PUFAs) have garnered interest for their multifaceted biological properties. These essential fatty acids particularly eicosapentaenoic acid (EPA) and docosahexaenoic acid (DHA) exert anti-inflammatory, antioxidant, and anti-apoptotic effects via modulation of membrane lipid composition, inhibition of Nuclear Factor-kappa B (NF-κB) signaling, and enhancement of endogenous antioxidant systems [4]. Moreover, OM3 fatty acids have been shown to stabilize mitochondrial membranes, suppress ROS production, and inhibit Cas activation, thus offering a plausible mechanism for mitochondria-centered protection in lung injury models [5].

Although previous studies have demonstrated the systemic benefits of OM3 fatty acids in critical illness, their role in preventing mitochondrial-driven apoptosis in the setting of LPS-induced lung injury remains to be fully elucidated [16, 19]. A deeper understanding of this relationship may reveal novel therapeutic avenues for halting the progression of sepsis-related lung dysfunction.

In this experimental study, we aimed to investigate the protective effects of OM3 fatty acids against LPS-induced acute lung injury, with a particular focus on the modulation of mitochondrial apoptotic signaling pathways. We hypothesized that OM3 fatty acids supplementation would ameliorate histopathological damage, reduce oxidative stress, suppress inflammatory cytokine expression, and attenuate mitochondrial apoptosis thereby preserving lung tissue integrity in an experimental sepsis model.

## Materials and methods

### Animals and experimental design

A total of 32 adult female Wistar albino rats (8–10 weeks old, 250–300 g) were procured from the Süleyman Demirel University Experimental Animal Laboratory. Animals were housed under controlled conditions (22 ± 2 °C, 12-h light/dark cycle, 55 ± 5% humidity) with ad libitum access to standard pellet diet and tap water. All experimental protocols were approved by the SDU Local Ethics Committee for Animal Experiments (Protocol No: 01.08.2024-09/319). Furthermore, the study was conducted and reported in compliance with the ARRIVE (Animal Research: Reporting of In Vivo Experiments) guidelines to ensure scientific rigor and ethical transparency. This study was supported by Süleyman Demirel University Scientific Research Project Unit with TTU-2024-9539 project number. All rats were randomly divided into four experimental groups (n = 8 per group):

1. *Control (Sham)* Received 0.3 ml of 0.9% saline intraperitoneally (ip) for 3 days and one additional at the third day.
2. *LPS* Rats received 0.9% saline ip for 3 days and a single ip 5 mg/kg LPS (Escherichia coli O55:B5; Sigma-Aldrich, Sweden) at third day to induce ALI [18].
3. *LPS + Omega-3 (LPS-OM3)* LPS was administered as described above, followed 30 min later by the last dose of OM3 (Omegaven®, Fresenius Kabi, Istanbul, Türkiye). OM3 was given ip at 400 mg/kg/day for 3 consecutive days [8].
4. *Omega-3 only (OM3)* Received only ip OM3 at the same dose and duration.

Rats in the LPS-OM3 and OM3 groups received OM3 (Omegaven® 10%, Fresenius Kabi, Istanbul, Türkiye) at a dose of 400 mg/kg/day (approximately 1.0–1.2 mL depending on body weight) administered intraperitoneally for 3 consecutive days. Omegaven® is a 10% fish oil emulsion containing 100 mg of fish oil per mL, consisting of eicosapentaenoic acid (EPA, 1.25–2.82 g/100 mL) and docosahexaenoic acid (DHA, 1.44–3.09 g/100 mL). Saline (0.9% NaCl) was utilized as the vehicle control for both Sham and LPS groups. Although OM3 is an emulsion, saline was preferred over an oily vehicle to prevent potential confounding effects from other bioactive lipids, ensuring that the observed changes were specifically attributable to OM3 supplementation.

At the end of the experimental period (6 h post LPS injection), rats were anesthetized with 80 mg/kg ketamine (Keta-Control, Doğa İlaç, Türkiye) and 10 mg/kg xylazine (XylazinBio, Bioveta, Czech Republic) and blood and lung tissues were harvested for further analyses.

### Histopathological and immunohistochemical analyses

Lung tissues were fixed in 10% buffered formalin for 48–72 h, processed routinely, and embedded in paraffin. Sections of 5 µm thickness were stained with hematoxylin–eosin (H&E) for histological evaluation.

All stained sections were examined and photographed using a Nikon Eclipse E-600 microscope (Nikon, Japan) equipped with the NIS-Elements imaging analysis system (Nikon, Japan). Histopathological changes were evaluated in 20 randomly selected fields at 400 × magnification. For the assessment of staining, alveolar and interstitial inflammation, edema, alveolar and interstitial hemorrhage, hyaline membrane formation, and epithelial detachment and disruption were scored using the Smith scoring system on a scale of 0–4 (0: no damage; 1: damage in 25% of the area; 2: damage in 50% of the area; 3: damage in 75% of the area; 4: damage in the entire area) [13].

For immunohistochemical analysis, 5 µm sections were obtained from paraffin-embedded blocks, mounted on poly-L-lysine-coated slides, and dried at 45 °C overnight. Sections were deparaffinized, rehydrated through a graded ethanol series, and subjected to antigen retrieval in citrate buffer (pH 6) by microwaving (2 × 5 min). Endogenous peroxidase was blocked with H₂O₂, and sections were incubated with Ultra V Block, followed by primary antibodies for TNF-α (1:100), Caspase-3 (1:200), and HSP-70 (1:200) at room temperature for 60 min. After washing, biotinylated secondary antibody and streptavidin-HRP were applied, and staining was visualized using DAB with hematoxylin counterstain. Slides were examined and photographed with a Nikon Eclipse E-600 microscope equipped with the NIS-Elements system. Twenty randomly selected fields at 400 × magnification were evaluated for staining intensity, which was assessed semiquantitatively by two independent observers who were blinded to the experimental groups on a 0–3 scale (0: no staining; 1 + : weak; 2 + : moderate; 3 + : strong) [17].

### Biochemical analysis

Lung tissues were homogenized in phosphate-buffered saline (PBS, pH 7.4) using an IKA Ultra-Turrax homogenizer. Supernatants were collected after centrifugation at 5000 rpm for 15 min at 4 °C. Oxidative stress parameters were determined spectrophotometrically:

- Total Antioxidant Status (TAS) and Total Oxidant Status (TOS) were measured using commercial kits (Rel Assay Diagnostics, Türkiye).
- Oxidative Stress Index (OSI) was calculated as: OSI = (TOS / TAS) × 100.

Protein concentrations were standardized using the Bradford method [2].

### qRT-PCR analysis

Total RNA was extracted from lung tissues using TRIzol reagent (Invitrogen, USA), and cDNA synthesis was performed using a High-Capacity cDNA Reverse Transcription Kit (Thermo Fisher Scientific).

qRT-PCR was carried out using SYBR Green Master Mix (Roche, Germany) and gene-specific primers for Bcl-2, Bax, Cyt-c, and Cas-9, with GAPDH as the housekeeping gene. Reactions were run on a BioRad CFX 96 System. Relative gene expression was calculated using the 2^−ΔΔCt^ method [14]. Primary sequences or genes are displayed in Table 1.

**Table 1:** Primer sequences, product sizes, and accession numbers of target genes used in qRT-PCR analysis

Genes	Specific primer sequence Forward (F)-Reverse (R)	Product size (bp)	Accession number
Bcl-2	F: CATCTCATGCCAAGGGGGAA	284 bp	NM_016993.2
	R: TATCCCACTCGTAGCCCCTC		
Bax	F. CACGTCTGCGGGGAGTCAC	419 bp	NM_017059.2
	R: TAGAAAAGGGCAACCACCCG		
Cyt-c	F: TAAATATGAGGGTGTCGC	192 bp	NM_012839.2
	R: AAGAATAGTTCCGTCCTG		
Cas-9	F: AGCCAGATGCTGTCCCATAC	148 bp	XM_039110693.1
	R: CAGGAACCGCTCTTCTTGTC		
GAPDH (HouseKeeping)	F: AGGTTGTCTCCTGTGACTTC	130 bp	NM_017008.4
	R: CTGTTGCTGTAGCCATATTC		

Bcl-2: B-cell lymphoma 2, Bax: Bcl-2-associated X protein, Cyt-c: Cytochrome c, Cas-9: Caspase-9

### Statistical analysis

Prior to parametric analysis, the normality of the data distribution was confirmed using the Shapiro–Wilk test, and the homogeneity of variances was verified with Levene’s test. All data utilized for One-way ANOVA followed by the Bonferroni post-hoc test were confirmed to meet these statistical assumptions.

Data were analyzed using GraphPad Prism 9.0. All values are presented as mean ± standard deviation (SD). One-way ANOVA followed by Bonferroni multiple comparison test was used for intergroup comparisons. A *p* value < 0.05 was considered statistically significant.

## Results

### Biochemical analysis

LPS-induced lung injury significantly altered the oxidative balance as reflected by changes in TOS, TAS, and OSI parameters. TOS levels were markedly increased in the LPS group compared to the control group (*p* = 0.025), indicating elevated oxidative stress. Notably, it was also higher compared to OM3 group as well. TAS levels in the LPS-OM3 group were numerically higher than those in the LPS group; however, this difference was not statistically significant (*p* > 0.05).

**Fig. 1:**
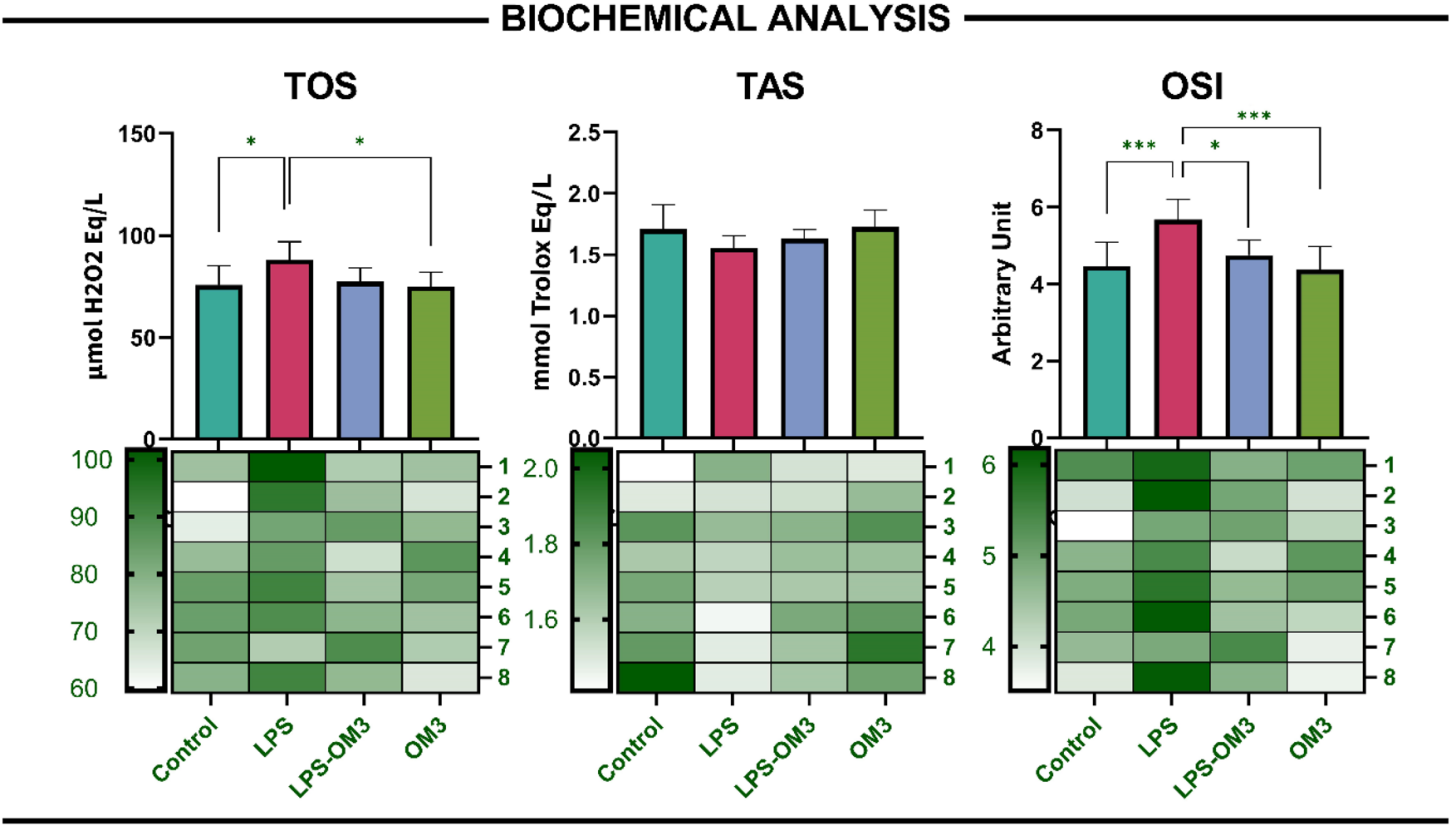
Biochemical evaluation of oxidative stress parameters in lung tissue. Bar graphs and corresponding heatmaps illustrate Total Oxidant Status (TOS), Total Antioxidant Status (TAS), and Oxidative Stress Index (OSI) levels in rat lung tissues across four experimental groups: Control, LPS, LPS-OM3 (omega-3), and OM3. TOS: LPS significantly increased oxidant levels vs. control (*p = 0.025); OM3 group showed lower levels vs LPS group (*p = 0.016). TAS: No statistically significant differences were found among groups. OSI: LPS induced a significant increase in OSI vs. control (***p < 0.001); this effect was significantly reversed by omega-3 both in LPS-OM3 and OM3 groups (*p = 0.014, ***p < 0.001 respectively). Data are presented as mean ± SEM (n = 8 per group). Heatmaps below each graph display normalized values per subject

OSI, calculated as the TOS/TAS ratio, was significantly elevated in the LPS group compared to the control group (*p* < 0.001), confirming the presence of an oxidative shift. Importantly, OM3 administration effectively attenuated the OSI increase, as evidenced by significant reductions in both the LPS-OM3 and OM3 groups relative to the LPS group (*p* = 0.014, *p* < 0.001 respectively) (Fig. 1).

### Histopathological analysis

Histopathological scoring of lung tissues revealed that LPS administration induced severe structural damage and inflammation, while OM3 pre-treatment significantly alleviated these alterations.

In the separation and disruption of the bronchiole epithelium, LPS group displayed significantly higher scores compared to the control (*p* < 0.001). Pre-administration of OM3 (LPS-OM3) markedly reduced epithelial damage relative to LPS alone (*p* < 0.018), though scores remained slightly elevated compared to control (*p* < 0.001).

Alveolar and interstitial inflammation was significantly intensified by LPS exposure (*p* < 0.001 vs. control), whereas LPS-OM3 treatment significantly ameliorated inflammatory cell infiltration (*p* < 0.001 vs. LPS). The OM3 group showed no significant inflammation, comparable to control.

For alveolar and interstitial hemorrhage, LPS group exhibited significantly elevated scores compared to all other groups (*p* < 0.001 for all). OM3 treatment substantially reduced hemorrhagic damage in the LPS-OM3 group (*p* < 0.001), indicating a potent protective effect.

Likewise, hyaline membrane formation, a hallmark of acute lung injury, was observed prominently in the LPS group but was significantly diminished in the LPS-OM3 group (*p* < 0.001). Both control and OM3 groups showed negligible membrane formation.

**Fig. 2:**
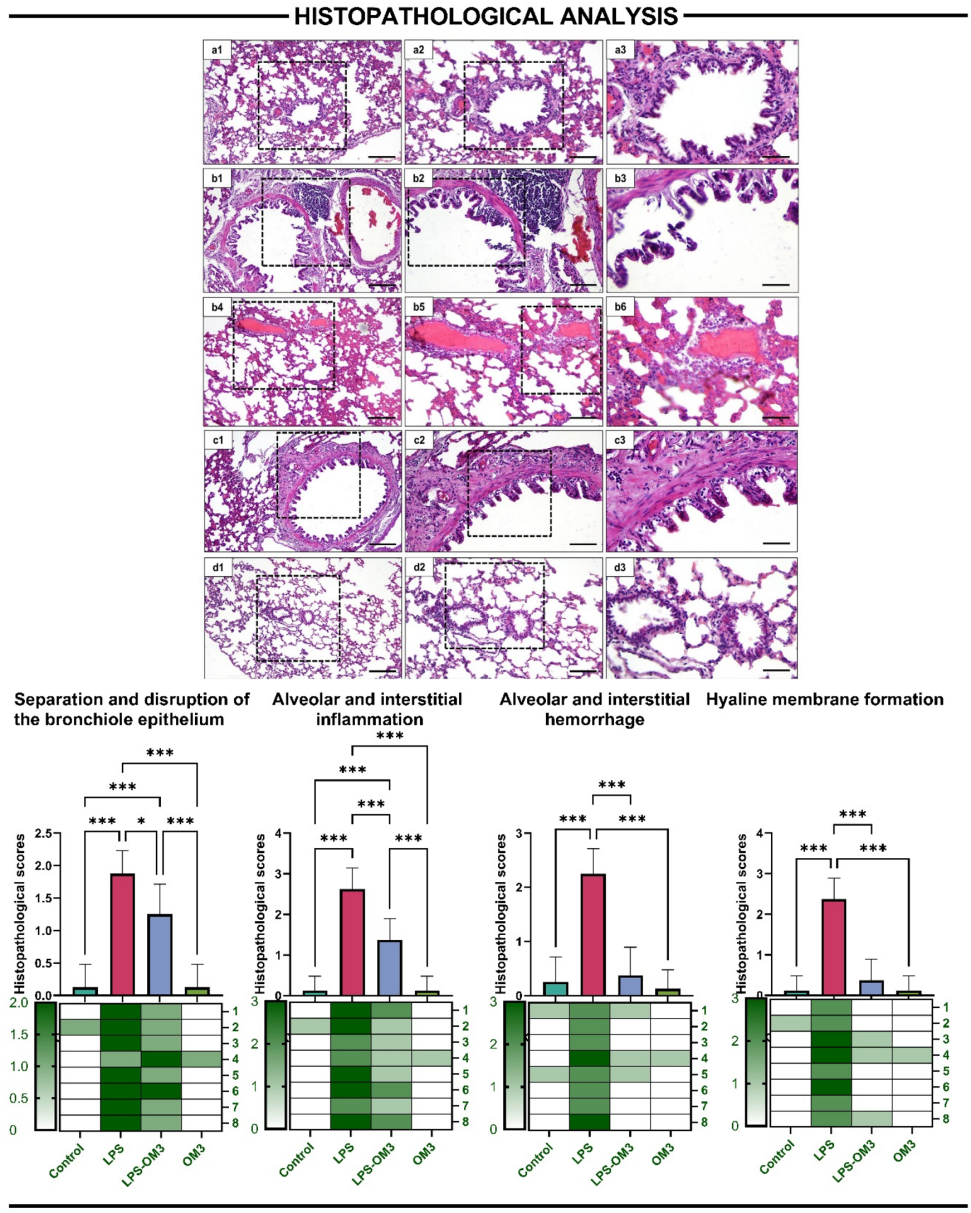
Representative histological images (H&E staining) and semi-quantitative scoring results demonstrating the extent of lung injury in different experimental groups. **a1**–**a3** Control group: Normal pulmonary architecture with intact bronchiolar epithelium, clear alveolar spaces, and absence of inflammation or hemorrhage, **b1**–**b6** LPS group: Severe epithelial disruption, extensive alveolar and interstitial inflammation, hemorrhage, and hyaline membrane formation, **c1**–**c3** LPS-OM3 group: Partial improvement in lung structure, with reduced epithelial separation, inflammation, and hemorrhage compared to LPS alone, **d1**–**d3** OM3 group: Preserved lung architecture similar to the control. Bar graphs represent the semi-quantitative histopathological scores for: Separation and disruption of the bronchiolar epithelium, Alveolar and interstitial inflammation, Alveolar and interstitial hemorrhage, Hyaline membrane formation. Significant histological deterioration was observed in the LPS group, which was significantly ameliorated by OM3 administration. Data are presented as mean ± SD; *p < 0.05 ***p < 0.001

Overall, OM3 pre-treatment significantly reduced the histological severity of LPS-induced lung injury, supporting its protective and anti-inflammatory effects (Fig. 2).

### Immunohistochemical analysis

**Fig. 3:**
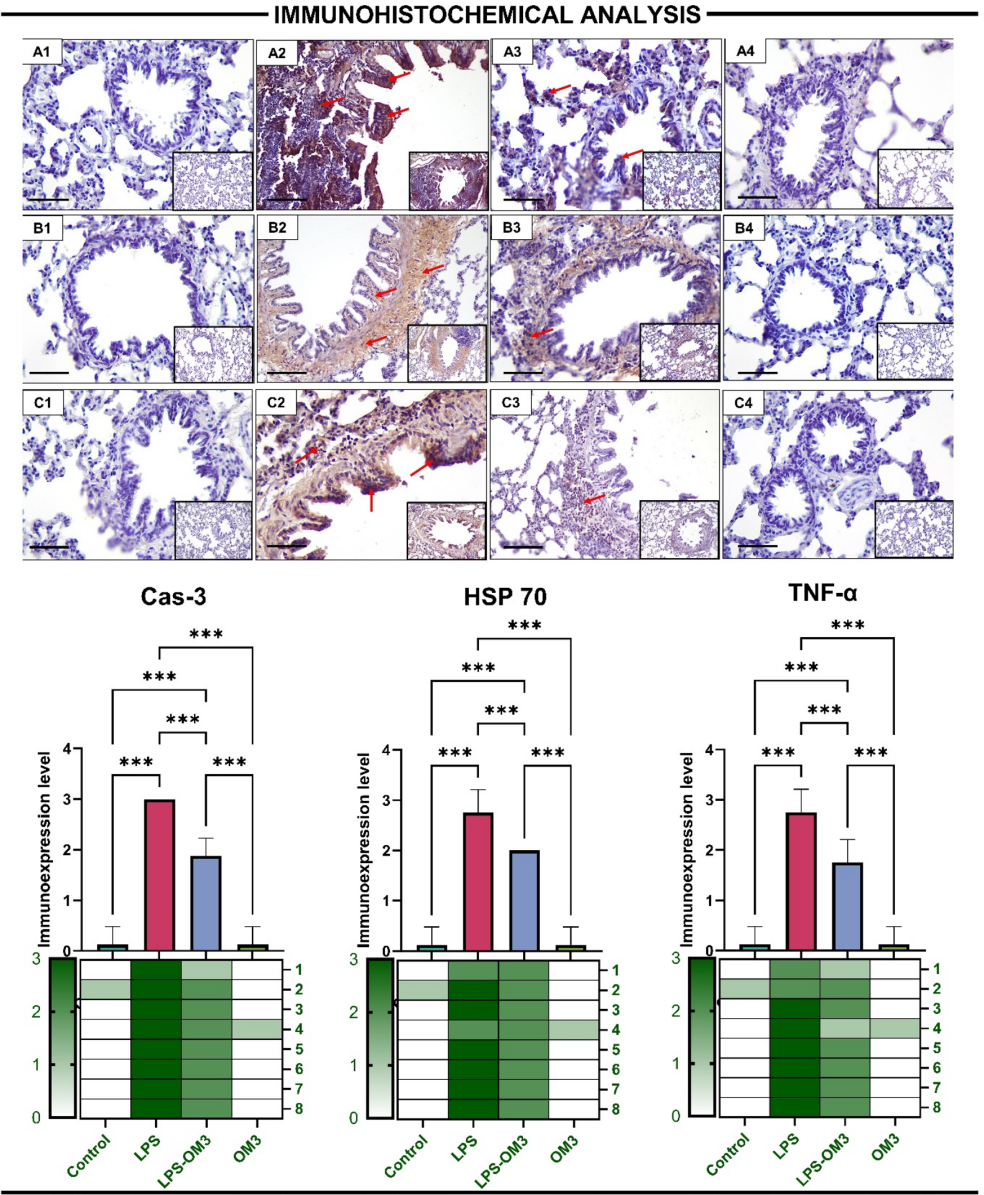
Representative immunohistochemical images and semi-quantitative scoring of TNF-α, HSP70, and Caspase-3 expression in lung tissues of rats from different experimental groups. **A1**–**A4** (Caspase-3). **A1** Control group—nearly absent Cas-3 positivity, **A2** LPS group—strong Cas-3 staining in bronchiolar and alveolar regions (red arrows), indicating apoptosis. **A3** LPS-OM3 group—reduced expression. **A4** OM3 group—similar to control. **B1**–**B4** (HSP70): **B1** Control group—very low HSP70 expression, **B2** LPS group—intense cytoplasmic HSP70 positivity in bronchial epithelial and alveolar cells (red arrows), **B3** LPS-OM3 group—moderate HSP70 expression, **B4** OM3 group—minimal expression, C1–C4 (TNF-α): **C1** Control group—minimal TNF-α expression, **C2** LPS group—marked TNF-α immunoreactivity, especially in bronchiolar epithelium and alveolar septa (red arrows), **C3** LPS-OM3 group—reduced TNF-α staining intensity, **C4** OM3 group—expression comparable to control. Bar graphs show semi-quantitative immunohistochemical expression scores for: Cas-3 (apoptosis executor protein), HSP70 (stress-related chaperone), TNF-α (inflammatory cytokine), LPS exposure significantly elevated the expression of all three markers, reflecting inflammation, cellular stress, and apoptosis, respectively. OM3 treatment significantly mitigated these increases. Data are presented as mean ± SD; ***p < 0.001

Immunohistochemical evaluation of lung tissues revealed that LPS administration significantly upregulated the expression of pro-inflammatory and stress-related proteins, while OM3 supplementation attenuated these elevations (Fig. 3).

Cas-3, a key executioner of apoptosis, showed strong immunopositivity in LPS-treated lungs (*p* < 0.001 vs. control). This apoptotic response was significantly reduced by OM3 pre-administration (*p* < 0.001 vs. LPS), indicating its anti-apoptotic action in the context of inflammatory lung injury.

Similarly, HSP70, a heat shock protein indicative of cellular stress, was significantly overexpressed in the LPS group (*p* < 0.001 vs. control). OM3 administration significantly mitigated this increase in the LPS-OM3 group (*p* < 0.001 vs. LPS), suggesting a protective effect against LPS-induced proteotoxic stress.

Furthermore, TNF-α expression was markedly increased in the LPS group compared to control (*p* < 0.001), indicating robust activation of the inflammatory cascade. Treatment with OM3 significantly reduced TNF-α immunoreactivity compared to the LPS group (*p* < 0.001), though levels remained slightly higher than control. The OM3 group showed minimal staining, similar to the control group.

Collectively, these findings highlight the modulatory effects of OM3 on inflammation, stress response, and apoptotic pathways following LPS-induced lung injury.

### qRT-PCR analysis

**Fig. 4:**
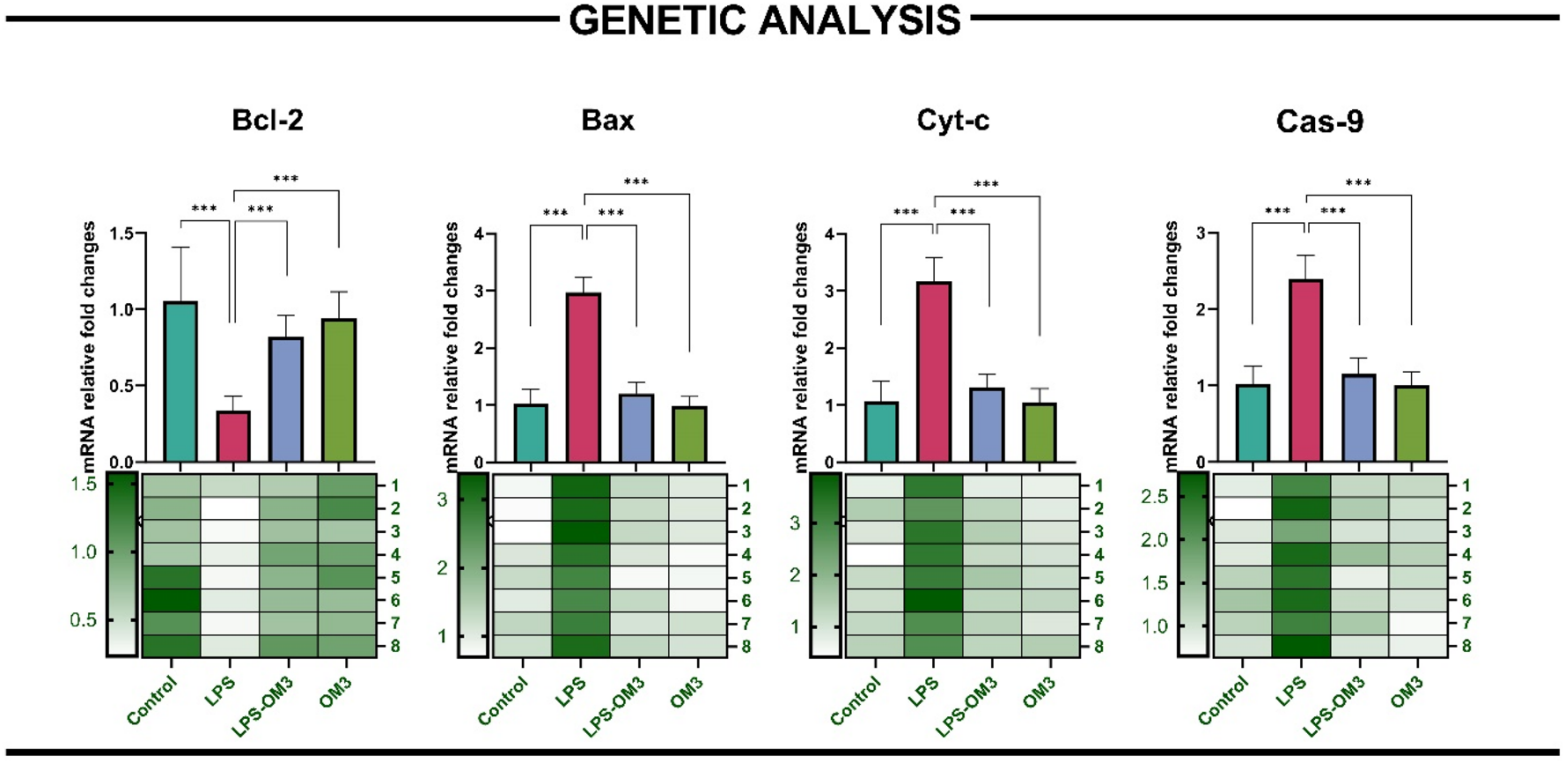
mRNA expression levels of apoptosis-related genes in lung tissue assessed by qRT-PCR. Bar graphs and heatmaps depict relative fold changes (mean ± SEM, n = 8) in: Bcl-2: LPS group showed significant downregulation (***p < 0.001 vs. control); LPS-OM3 group showed significant recovery (***p < 0.001 vs. LPS). Bax: Markedly increased in LPS group (***p < 0.001); significantly reduced in LPS-OM3 (***p < 0.001 vs. LPS). Cyt-c and Cas-9: Both significantly upregulated in LPS group (***p < 0.001), with OM3 reversing these elevations in the LPS-OM3 group (***p < 0.001 for both comparisons). Heatmaps below each gene panel illustrate individual animal values, highlighting consistent regulatory effects across subjects

Quantitative real-time PCR analysis revealed significant alterations in the expression of key apoptotic genes following LPS-induced lung injury, and OM3 pre-treatment effectively modulated these changes (Fig. 4).

Bcl-2, an anti-apoptotic gene, was significantly downregulated in the LPS group compared to control (*p* < 0.001), indicating impaired cell survival signaling. However, its expression was markedly restored in the LPS-OM3 group (*p* < 0.001 vs. LPS), suggesting a protective effect of OM3 against apoptotic cell loss.

In contrast, pro-apoptotic Bax levels were significantly elevated in the LPS group (*p* < 0.001 vs. control). Pre-administration of OM3 significantly reduced Bax expression in the LPS-OM3 group (*p* < 0.001 vs. LPS), indicating suppression of pro-death signaling.

Similarly, Cyt-c expression, reflecting mitochondrial outer membrane permeabilization and intrinsic apoptotic activation, was dramatically increased in the LPS group (*p* < 0.001), while the LPS-OM3 group showed significantly attenuated expression (*p* < 0.001 vs. LPS).

Cas-9, an initiator caspase in the intrinsic apoptosis pathway, also showed significant upregulation following LPS exposure (*p* < 0.001), which was significantly reversed by OM3 treatment (*p* < 0.001 vs. LPS).

These results collectively demonstrate that OM3 effectively modulates the intrinsic apoptotic pathway by promoting anti-apoptotic Bcl-2 and suppressing Bax, Cyt-c, and Cas-9 expression, thereby mitigating LPS-induced mitochondrial apoptosis in lung tissue.

## Discussion

LPS-induced ALI is characterized by excessive inflammation, oxidative stress, and structural damage, ultimately leading to alveolar-capillary barrier dysfunction and respiratory failure. One of the key pathological events implicated in ALI is mitochondrial apoptosis, which amplifies tissue injury through caspase cascade activation and loss of epithelial integrity [15]. The present study provides compelling evidence that OM3 fatty acids exert a robust protective effect against LPS-induced pulmonary damage by modulating mitochondrial apoptotic signaling, alleviating oxidative stress, and attenuating inflammation.

Histopathological evaluations clearly demonstrated severe lung tissue alterations following LPS administration, including bronchiolar epithelial disruption, alveolar hemorrhage, interstitial inflammation, and hyaline membrane formation. These histological lesions are consistent with previous reports highlighting LPS-induced neutrophilic infiltration and endothelial injury [15]. Importantly, OM3 pre-treatment significantly mitigated all histological damage parameters, indicating its potent structural protective capacity.

One of the key findings of this study is the reduction in oxidative stress markers in the OM3-treated groups. TOS levels were significantly elevated in the LPS group, reflecting excessive ROS generation, which is a hallmark of ALI ([12]). Concurrently, OSI values increased while TAS remained unchanged, suggesting a shift towards a pro-oxidant state. OM3 supplementation reversed these trends by significantly decreasing OSI. This antioxidant effect of OM3 is likely mediated through its membrane-stabilizing actions and ability to enhance endogenous antioxidant enzymes such as glutathione peroxidase [4].

The reduction in oxidative burden is mechanistically relevant to mitochondrial integrity, as mitochondrial membranes are highly susceptible to ROS-induced damage. This link is further corroborated by our gene expression data, wherein LPS exposure caused a profound increase in Bax, Cyt-c, and Cas-9, alongside a suppression of Bcl-2. These genes are central components of the intrinsic (mitochondrial) apoptotic pathway, and their dysregulation suggests initiation of mitochondrial outer membrane permeabilization, Cyt-c release, and apoptosome formation [9, 23]. Notably, OM3 pre-administration normalized these expressions, rebalancing pro- and anti-apoptotic signaling.

Parallel to the genetic data, immunohistochemical staining of Cas-3, the final executor caspase, was markedly elevated in the LPS group and significantly suppressed by OM3. This confirms that upstream mitochondrial signaling alterations translated into functional execution of apoptosis. The observed Cas-3 inhibition by OM3 supports its anti-apoptotic action and aligns with previous findings demonstrating OM3’s role in suppressing apoptosis in various inflammatory models [7, 10, 22].

Further reinforcing these findings, TNF-α, a key pro-inflammatory cytokine that can also activate apoptosis through mitochondrial pathways, was significantly elevated in the LPS group and suppressed by OM3. TNF-α has been shown to induce mitochondrial ROS production and Bax translocation [21]. Hence, its downregulation by OM3 likely contributes to both reduced inflammation and suggests mitochondrial stabilization.

Another critical stress marker, HSP70, was markedly upregulated in the LPS group, suggesting cellular attempts to mitigate protein misfolding and mitochondrial stress. OM3 significantly attenuated HSP70 expression, reflecting reduced cellular stress and protein damage. Since HSP70 can also bind Apaf-1 and inhibit apoptosome formation [3]. Its modulation is likely to have downstream consequences on caspase activation.

These biochemical and immunohistochemical observations are well-aligned with the histological outcomes, whereby OM3 prevented epithelial disintegration and hyaline membrane formation events commonly attributed to massive epithelial cell death via apoptosis [15]. Thus, it is evident that the attenuation of mitochondrial apoptotic signaling by OM3 translates into tangible preservation of tissue architecture.

Interestingly, the OM3-only group did not differ significantly from control in any parameter, indicating its safety and lack of pro-inflammatory or pro-apoptotic effects under physiological conditions. This suggests OM3 may act specifically under pathological stimuli to restore homeostasis.

The integrated interpretation of all data supports the hypothesis that OM3 exerts its protective effect primarily by inhibiting mitochondrial apoptosis, which represents the final convergence point of inflammation and oxidative injury. This multifaceted regulation is particularly relevant in LPS-induced ALI, where mitochondrial dysfunction is central to disease progression.

Moreover, our findings provide a mechanistic explanation for clinical observations reporting improved respiratory outcomes with OM3 supplementation in patients with sepsis and ARDS [6, 20]. By suppressing mitochondrial damage, OM3 not only prevents epithelial cell loss but also curtails inflammatory amplification loops perpetuated by apoptotic bodies and DAMPs.

Another important consideration is the role of mitochondrial ROS in linking oxidative stress with apoptotic signaling. The observed reductions in OSI and apoptotic gene expression suggest that OM3 may prevent MOMP increase by stabilizing mitochondrial membranes and reducing ROS leakage, as reported in cardiomyocyte models [1].

**Fig. 5:**
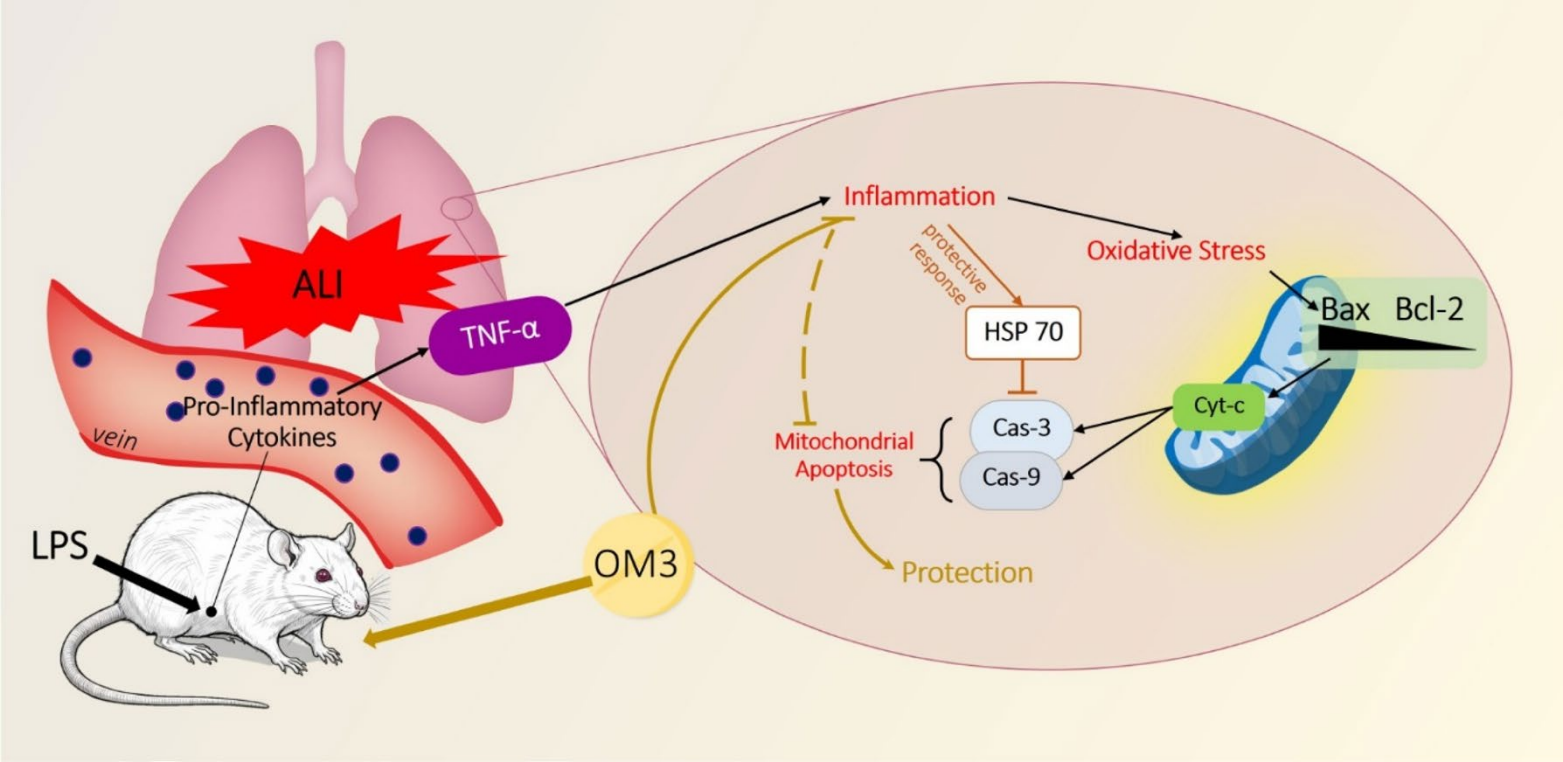
Schematic representation of the protective effects of OM3 fatty acids against LPS-induced acute lung injury in rats. LPS exposure triggers inflammation, oxidative stress, mitochondrial apoptosis, and structural lung damage, evidenced by elevated Bax, Cyt-c, Cas-9 expression, increased TNF-α, HSP70, and Cas-3 levels, as well as severe histopathological alterations such as epithelial disruption, hemorrhage, inflammation, and hyaline membrane formation. OM3 supplementation mitigates these effects by downregulating apoptotic and pro-inflammatory markers (Bax, Cyt-c, Cas-9, TNF-α, Cas-3), restoring Bcl-2 expression, and improving tissue integrity and antioxidant capacity. This multi-pathway modulation highlights the therapeutic potential of OM3 in preventing mitochondrial dysfunction-mediated pulmonary injury

Taken together, these data highlight the central role of mitochondria as both a target and mediator in LPS-induced lung injury, and suggest OM3 as a promising mitochondrial-protective agent. This is particularly significant given the limited efficacy of current therapies targeting inflammation or oxidative stress in isolation. The integrated interpretation of all data supports the hypothesis that OM3 exerts its protective effect primarily by inhibiting mitochondrial apoptosis (Fig. 5).

Our findings align with the existing literature emphasizing the protective properties of OM3 fatty acids against sepsis-induced lung injury. Specifically, Zhu et al. demonstrated that alpha-linolenic acid, an n-3 PUFA, exerts significant protection against septic lung damage through anti-inflammatory and anti-oxidative signaling pathways [25]. While these studies predominantly focus on the inflammatory cascade and systemic oxidative stress, our results contribute to the literature by exploring the potential molecular mechanisms associated with mitochondrial preservation. Our findings suggest that OM3 supplementation may provide lung protection, potentially by supporting mitochondrial integrity and modulating intrinsic apoptotic pathways, as indicated by the favorable changes in the Bax/Bcl-2 ratio and Caspase-9 expression. Furthermore, research by Kocherlakota et al. supports the efficacy of OM3 fatty acids in maintaining pulmonary architecture and tissue integrity under lipopolysaccharide (LPS)-induced stress [n^11^]. These parallels reinforce our hypothesis that OM3 supplementation serves as a multifaceted protective strategy, particularly through its regulatory role at the mitochondrial level.

Despite its significant findings, this study has several limitations. First, the lack of mitochondrial ultrastructural analysis (via TEM) and protein-level validation (via Western blot) means the evidence for mitochondrial preservation remains primarily based on biochemical and gene expression data. Second, the use of exclusively female rats and a single dose of omega-3 (400 mg/kg) limits the generalizability of the results and precludes the assessment of a dose–response relationship. Finally, the absence of a positive control group, such as dexamethasone, prevents a direct comparison of omega-3’s efficacy against established anti-inflammatory treatments. Future research addressing these factors is necessary to further validate the clinical potential of omega-3 in septic lung injury."

Despite these limitations, the consistent pattern across biochemical, histological, immunohistochemical, and genetic datasets supports a unified conclusion: OM3 fatty acids significantly attenuate LPS-induced lung injury by mitigating oxidative stress, reducing inflammation, and most importantly suppressing mitochondrial apoptotic signaling.

## Data Availability

All data revelant to this research is available upon request from corresponding Author.
